# Sudden cardiac death after alcohol intake: classification and autopsy findings

**DOI:** 10.1038/s41598-022-20250-3

**Published:** 2022-10-06

**Authors:** Lauri Holmström, Janna Kauppila, Juha Vähätalo, Lasse Pakanen, Juha Perkiömäki, Heikki Huikuri, Juhani Junttila

**Affiliations:** 1 Research Unit of Internal Medicine, Medical Research Center Oulu, University of Oulu and Oulu University Hospital, PO Box 5000, 90014 Oulu, Finland; 2 Forensic Medicine Unit, Finnish Institute for Health and Welfare (THL), Oulu, Finland; 3 Department of Forensic Medicine, Research Unit of Internal Medicine, Medical Research Center Oulu, University of Oulu, Oulu, Finland

**Keywords:** Cardiology, Medical research

## Abstract

Alcohol is known to have an immediate effect on cardiac rhythm, and previous studies have found that a notable proportion of sudden cardiac deaths (SCD) occur after alcohol intake. The objective of the present study was to investigate the association between the timing of alcohol intake and SCD. Our study population is drawn from the Fingesture study, which includes 5869 consecutive SCD cases from Northern Finland who underwent medicolegal autopsy 1998–2017. Toxicological analysis was performed if there was any suspicion of toxic exposure, or if there was no obvious immediate cause of SCD at autopsy. We found that 1563 (27%) of all SCD victims had alcohol in blood or urine at autopsy (mean age (61 ± 10 years, 88% male). Eighty-six percent of alcohol-related SCD victims had higher urine alcohol concentration than blood alcohol concentration, referring to the late-stage inebriation. These results suggest that the majority of alcohol-related SCDs occur at the late stage of inebriation.

## Introduction

Alcohol is one of the most consumed beverages worldwide, yet it is accountable for a significant proportion of disability-adjusted life-years due to various cardiac and noncardiac conditions. Cardiac arrhythmias and sudden cardiac death (SCD), the most common mode of death in Western Societies, are known to be provoked by binge drinking, but the relationship between the timing of alcohol consumption and SCD is unclear^1–3^. We aimed to investigate the proportions and characteristics of SCDs occurring in the early and late stages of alcohol inebriation.

## Methods

The study population is drawn from the Fingesture study, which has prospectively collected data from all SCDs in Northern Finland (population≈600,000) since 1998^1,2,5^. The study rationale is based on the Finnish law, which requires a medicolegal autopsy to be performed if the death is not due to a known disease, the victim has not been treated during his/her last illness, or the death is otherwise unexpected. SCD was defined as witnessed death within 6 h of the onset of the symptoms and as unwitnessed death, within 24 h of the time the victim was last seen in a stable state of health, and the death was determined to be due to cardiac disease in the subsequent medicolegal autopsy. Subjects in whom autopsy revealed non-cardiac causes of sudden death (e.g., cerebral hemorrhage, pulmonary embolism, non-natural causes) were not included in the Fingesture study. Consequently, the Fingesture study includes 5869 consecutive sudden deaths with autopsy-verified cardiac origin from Northern Finland during 1998–2017. Each autopsy was performed in the Finnish Institute for Health and Welfare, Oulu, Finland, and at the Department of Forensic Medicine, University of Oulu, Oulu, Finland, by an experienced forensic pathologist, each performing more than 100 autopsies per year and using contemporary guidelines for the diagnosis of the cause of death. Due to the basis of Finnish legislation, medicolegal autopsies have unified investigation protocol including meticulous cardiac examinations: macroscopic dissection and investigation of myocardium, valves and coronary arteries, and histological samples taken from 3 to 5 sections of the heart.

**Figure 1:**
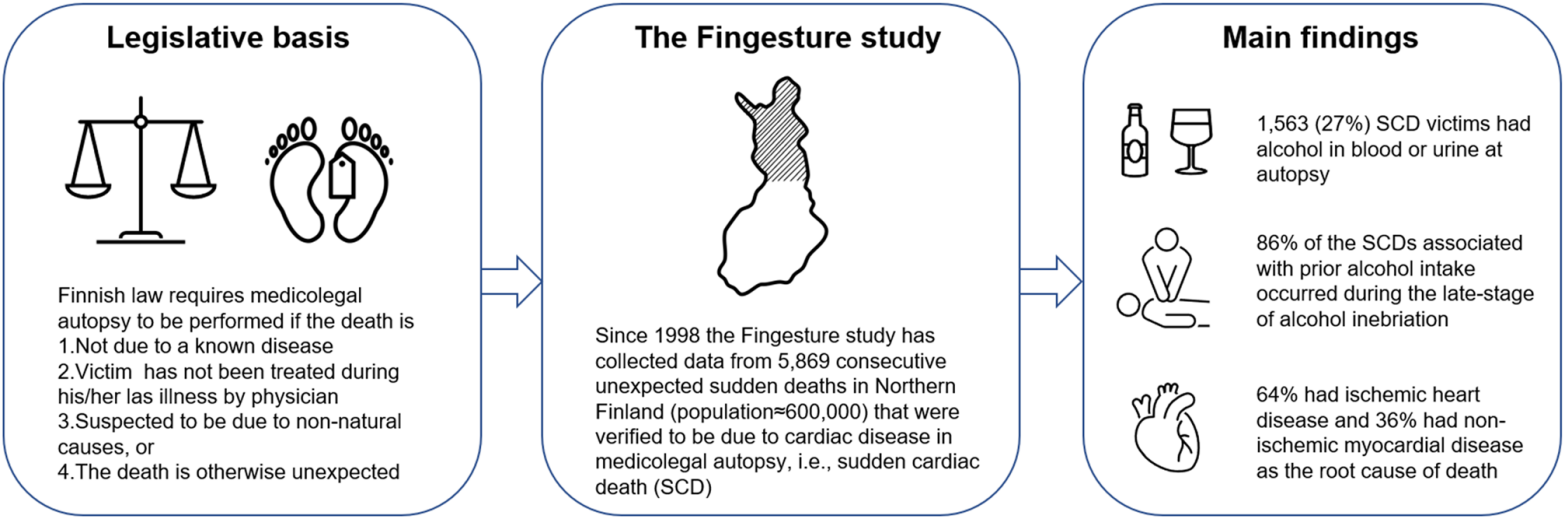
Schematic illustration of the study protocol and results.

Toxicological samples were collected, and analysis was performed after the decision by the forensic pathologist if there was any suspicion of toxic exposure prior to SCD, or if there was no obvious immediate cause of death at autopsy (e.g., macroscopically visible acute myocardial infarction). A comprehensive toxicological analysis of urine and blood samples with a multi-technique approach was used. Toxicological samples underwent screening and quantification analysis for poisonous substances and legal/illicit drugs, after which the analysis results were sent to the forensic pathologist. Detailed methods of toxicological analysis have been described earlier^4^. After toxicological results, the forensic pathologist issued a death certificate. Determination of the primary cause of death was based on a combination of autopsy findings, clinical records, and police reports. In the present study, all the SCDs were determined to have a cardiac disease as the primary cause of death, while the preceding alcohol inebriation was considered as a contributing factor. Based on previously established blood and urine alcohol metabolism^5^, the early-stage of alcohol inebriation was defined as blood alcohol concentration (BAC) higher than urine alcohol concentration (UAC), and late-stage as BAC ≤ UAC at autopsy. Schematic illustration of study rationale and results are presented in Fig. 1.

Continuous variables are presented as mean ± SD. Comparison of continuous variables was performed with Student t-test, chi-square test was used for categorical variables. All reported *p* values are two-sided and values less than 0.05 were considered significant. Statistical analyses were performed with the Statistical Package for Social Studies version 24.0 (SPSS Inc, Chicago, IL).

The study complies with the Declaration of Helsinki, and the Ethics Committee of Northern Ostrobothnia Hospital District and the National Authority for Medicolegal Affairs (Valvira) approved the study. Finnish Institute for Health and Welfare, and Regional State Administrative Agency of Northern Finland approved the review of medicolegal autopsy data by the investigators. Consent from next of kin was waived by the Ethics Committee since according to Finnish law, medicolegal autopsy does not require consent.

## Results

Out of 5,869 SCDs in the Fingesture study, 1563 (27%) had alcohol in blood or urine at autopsy. Eighty-six percent of the SCDs associated with prior alcohol intake had BAC ≤ UAC, referring to the late-stage, whereas 14% had BAC > UAC, referring to the early-stage of inebriation.

Those who died during the late stage had higher blood (1.2 ± 0.9 vs. 1.0 ± 0.8; *p* < 0.001) and urine alcohol levels (1.8 ± 1.2 vs. 0.7 ± 0.8; *p* < 0.001), were younger (61 ± 10 vs. 64 ± 11; *p* < 0.001), more often male (89% vs. 83%; *p* = 0.01), had a lower prevalence of myocardial fibrosis (91.2% vs. 95.9%; p = 0.02), had a higher prevalence of fatty liver (85.8% vs. 77.6%; *p* = 0.003) and higher liver weight (1812 ± 707 g vs. 1613 ± 690 g; *p* < 0.001) compared to those who died during the early stage. Previously diagnosed cardiovascular disease was more common among those who died during the early stage of inebriation (48.4% vs. 38.4%; *p* = 0.007). The most common causes of death in alcohol-related SCDs were coronary artery disease (CAD) (63.7%), hypertensive myocardial disease (11.0%), alcoholic cardiomyopathy (9.5%), and obesity-related cardiomyopathy (8.4%), and the proportions of cardiac hypertrophy (70.1%) and myocardial fibrosis (91.9%) were high. A detailed comparison between early and late-stage SCD is presented in Table 1.

**Table 1:** Characteristics of sudden cardiac death victims with alcohol either in blood or urine at autopsy. * = Data available in 498 cases (43 in BAC>UAC group and 455 in BAC≤UAC group). ARVC = arrhythmogenic right ventricular cardiomyopathy.

Characteristics	Overall (n = 1,563)	BAC > UAC (n = 220, 14%)	BAC ≤ UAC (n = 1343, 86%)	*P* value
Blood alcohol level, ‰	1.2 ± 0.9	1.0 ± 0.8	1.2 ± 0.9	< 0.001
Urine alcohol level, ‰	1.7 ± 1.2	0.7 ± 0.8	1.8 ± 1.2	< 0.001
B-alc/U-alc	0.7 ± 0.4	1.4 ± 0.6	0.6 ± 0.3	< 0.001
Age, years	61 ± 10	64 ± 11	61 ± 10	< 0.001
Male, %	88.1	82.7	89.0	0.01
BMI, kg/m^2^	27.7 ± 6.7	28.2 ± 7.0	27.6 ± 6.5	0.19
**Cause of death, %**				
Coronary artery disease	63.7	66.4	63.3	0.41
Hypertensive myocardial disease	11.0	8.2	11.5	0.16
Alcoholic cardiomyopathy	9.5	8.2	9.8	0.54
Obesity cardiomyopathy	8.4	9.1	8.3	0.79
Primary myocardial fibrosis	3.5	4.5	3.4	0.43
Dilated cardiomyopathy	1.0	1.4	0.9	0.71
Valve disease	0.8	0.5	0.8	0.71
Hypertrophic cardiomyopathy	0.8	0.9	0.8	1.00
Myocarditis	0.6	0.5	0.7	1.00
Structurally normal heart	0.4	0.0	0.4	0.60
ARVC	0.2	0.5	0.1	0.37
**Autopsy findings, %**				
Cardiac hypertrophy	70.1	69.1	70.2	0.75
Myocardial fibrosis	91.9	95.9	91.2	0.02
Myocardial scars	31.1	30.9	31.1	0.15
Fatty liver	84.6	77.6	85.8	0.003
Liver cirrhosis	28.2	25.1	28.7	0.29
Liver weight, g	1758 ± 709	1613 ± 690	1812 ± 707	< 0.001
**Comorbidities, %**				
Diabetes	20.1	24.5	19.4	0.09
Cardiovascular disease	39.8	48.4	38.4	0.007
Prior myocardial infarction	6.1	6.8	6.0	0.75
Heart failure	6.4	7.8	6.2	0.44
Dyslipidemia	12.9	14.1	12.7	0.57
Time of death*, %				0.11
12 pm-6am	31.5	30.2	31.6	
6am-12 pm	21.3	9.3	22.4	
12 pm-6 pm	23.3	37.2	22.0	
6 pm-12am	23.3	23.3	23.3	
**Location, %**				0.82
Indoors	88.7	88.2	88.8	
Outdoors	11.3	11.8	11.2	
Death during witnessed physical exertion	9.6	12.0	9.3	0.33

Alcohol concentrations in blood and urine were measured from 3,470 SCD victims. In comparison to SCD cases without alcohol in blood or urine, alcohol related SCD victims were more often male (88.1% vs. 80.4%; *p* < 0.001) and had less often CAD as the cause of SCD (63.7% vs. 67.3%; *p* = 0.03) and had more often hypertensive myocardial disease (11.0% vs. 8.0%; *p* = 0.002) or alcoholic cardiomyopathy (9.5% vs. 6.2%; *p* < 0.001) as the cause of SCD. The prevalence of fatty liver (84.6% vs. 70.3%; *p* < 0.001) and liver cirrhosis (28.2% vs. 21.3%; *p* < 0.001) at autopsy was more common in alcohol related SCD. Alcohol-related SCD occurred more often at night and indoors and less often during physical exertion. Detailedcharacteristics of SCD victims with known blood and urine alcohol concentrations are presented in Table 2.

**Table 2:** Characteristics of sudden cardiac death victims with known blood and urine alcohol concentrations. * = Data available in 1,229 cases (498 in those with alcohol in blood or urine and 731 in those without alcohol in blood or urine). ARVC = arrhythmogenic right ventricular cardiomyopathy.

Characteristics	Overall (n = 3,470)	Alcohol in blood or urine (n = 1,563, 45%)	No alcohol in blood or urine (n = 1907, 55%)	*p* value
Age, years	61.5 ± 11.8	61.3 ± 10.5	61.7 ± 12.8	0.34
Male, %	83.9	88.1	80.4	< 0.001
BMI, kg/m^2^	27.7 ± 6.6	27.7 ± 6.6	27.7 ± 6.5	0.95
**Cause of death, %**				
Coronary artery disease	65.7	63.7	67.3	0.03
Hypertensive myocardial disease	9.3	11.0	8.0	0.002
Alcoholic cardiomyopathy	7.7	9.5	6.2	< 0.001
Obesity cardiomyopathy	8.3	8.4	8.1	0.74
Primary myocardial fibrosis	4.2	3.5	4.7	0.08
Dilated cardiomyopathy	1.1	1.0	1.2	0.58
Valve disease	1.2	0.8	1.5	0.05
Hypertrophic cardiomyopathy	0.8	0.8	0.7	0.74
Myocarditis	1.2	0.6	1.6	0.008
Structurally normal heart	0.4	0.4	0.4	0.94
ARVC	0.1	0.2	0.1	0.23
**Autopsy findings, %**				
Cardiac hypertrophy	70.4	70.1	70.7	0.71
Myocardial fibrosis	91.2	91.9	90.6	0.19
Myocardial scars	32.3	31.1	33.2	0.19
Fatty liver	76.8	84.6	70.3	< 0.001
Liver cirrhosis	24.4	28.2	21.3	< 0.001
Liver weight, g	1792 ± 678	1784 ± 708	1798 ± 653	0.55
**Comorbidities, %**				
Diabetes	20.2	20.1	20.3	0.93
Cardiovascular disease	39.6	39.8	39.5	0.89
Prior myocardial infarction	5.7	6.1	5.4	0.41
Heart failure	7.2	6.4	7.9	0.13
Dyslipidemia	13.0	12.9	13.1	0.84
Time of death*, %				< 0.001
12 pm-6am	26.1	31.5	22.4	
6am-12 pm	24.4	21.3	26.5	
12 pm-6 pm	27.3	23.3	30.0	
6 pm-12am	21.8	23.3	20.8	
**Location, %**				< 0.001
Indoors	86.4	88.7	84.4	
Outdoors	13.6	11.3	15.6	
Death during witnessed physical exertion	12.8	9.6	15.5	< 0.001

## Discussion

In this autopsy-based study of SCD victims, we found that more than every fourth had alcohol in either blood or urine at autopsy. Most of the cases had higher urine than blood alcohol levels, referring to the late stage of alcohol inebriation. Although a common paradigm indicates that great majority of the SCDs after middle age is due to CAD, less than two-thirds of alcohol related SCDs had evidence of CAD in the autopsy, and less than 10% had alcoholic cardiomyopathy, but, intriguingly, still more than 90% had myocardial fibrosis and 70% had cardiac hypertrophy. The most common causes of hypertrophy and fibrosis were CAD, hypertension, alcohol, and obesity. However, 3.5% had primary myocardial fibrosis, which is an especially common autopsy finding among young SCD victims^6,7^.

Given that myocardial structural abnormalities are known to create an anatomic substrate that can maintain lethal ventricular arrhythmias leading to SCD^8^, it is reasonable to hypothesize that alcohol intake may act as a trigger for lethal arrhythmias among those with pre-existing structural cardiac disease, either ischemic or non-ischemic. Previous studies have demonstrated that alcohol has a direct effect on cardiomyocyte electrophysiology^9^, but the arrhythmogenic potential of binge drinking may also be due to various other factors, e.g., concomitant tachycardia-induced ischemia, alcohol’s negative inotropic effect, sympathetic activation, vasodilation, metabolic alterations, or electrolyte disturbances^10^. Of note, alcohol has direct cardiotoxic effects^11^, and alcohol-induced myocyte death may also affect the development of anatomic substrate (i.e., myocardial fibrosis) for subsequent arrhythmias.

Interestingly, most of our alcohol related SCDs occurred during the late stage of alcohol inebriation. The association between alcohol intake and SCD seems to be somewhat analogous to alcohol intake and atrial fibrillation, which also occurs mostly several hours after alcohol intake or during the late stage of inebriation^12^, reinforcing the hypothesis that alcohol has the greatest arrhythmogenic potential during the late stage of inebriation. The exact mechanism of how alcohol increases the risk of arrhythmia specifically during the late stage of inebriation remains unclear. Given the differences in characteristics between the early and late-stage SCDs in our study, these events may have somewhat distinct risk factors and underlying pathophysiology. Higher BAC, higher liver weight, and higher prevalence of fatty liver despite somewhat lower BMI may denote that subjects whose death occurred during the late stage had heavier alcohol consumption history.

Probably the most important limitation of our study is the absence of a control population, and hence we cannot estimate the relative risk of SCD during the early and late stages of alcohol inebriation. However, given that a relatively high proportion of SCD cases had alcohol in blood/urine and that alcohol intake has well-established proarrhythmic effects, it is unlikely that alcohol would have been an innocent bystander in SCD.

In conclusion, a significant proportion of SCDs occur immediately after alcohol intake and most of these events occur at the late stage of inebriation (Fig. 1). It remains obscure, however, why SCDs at the early and late stages differ in certain characteristics, and further studies are warranted to recognize possible event-specific risk profiles for SCD. These findings provide valuable information on the role of alcohol intake as a trigger for SCD, which may have the potential to translate into improved prediction and prevention of alcohol related SCDs.

## Data Availability

The datasets generated and analysed during the current study are not publicly available due to potentially identifiable nature but are available from the corresponding author on reasonable request.
